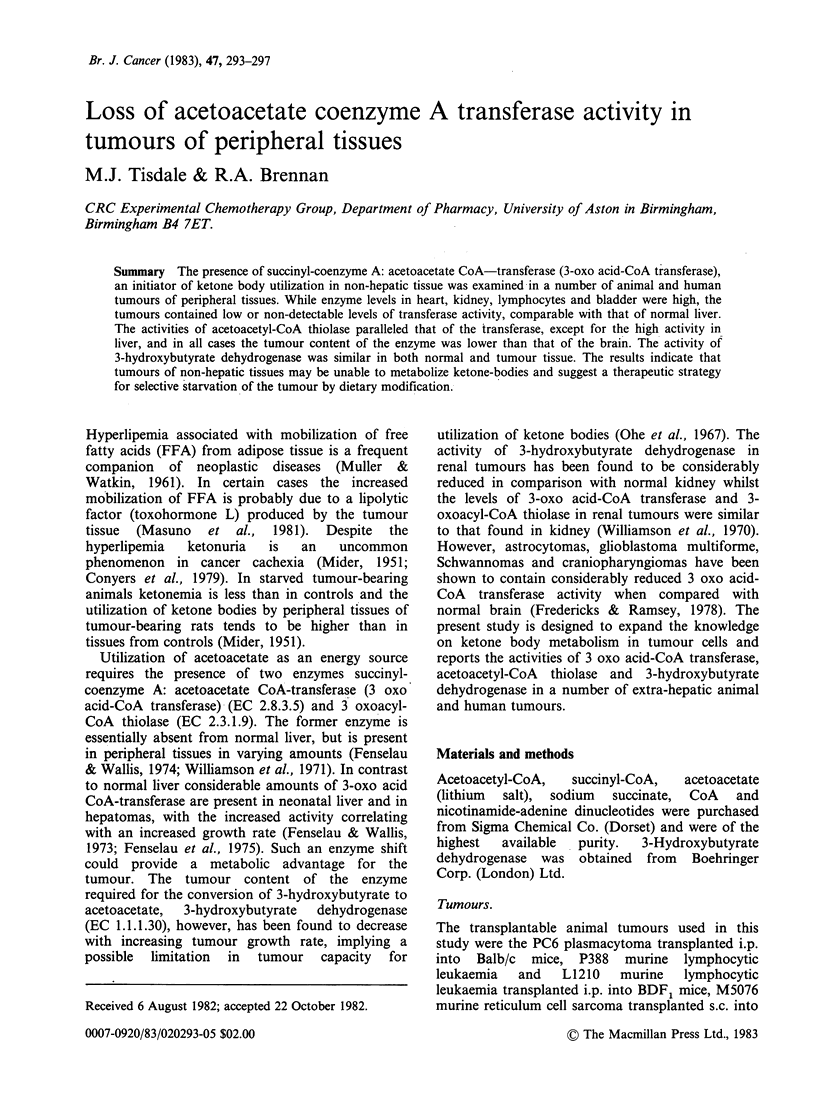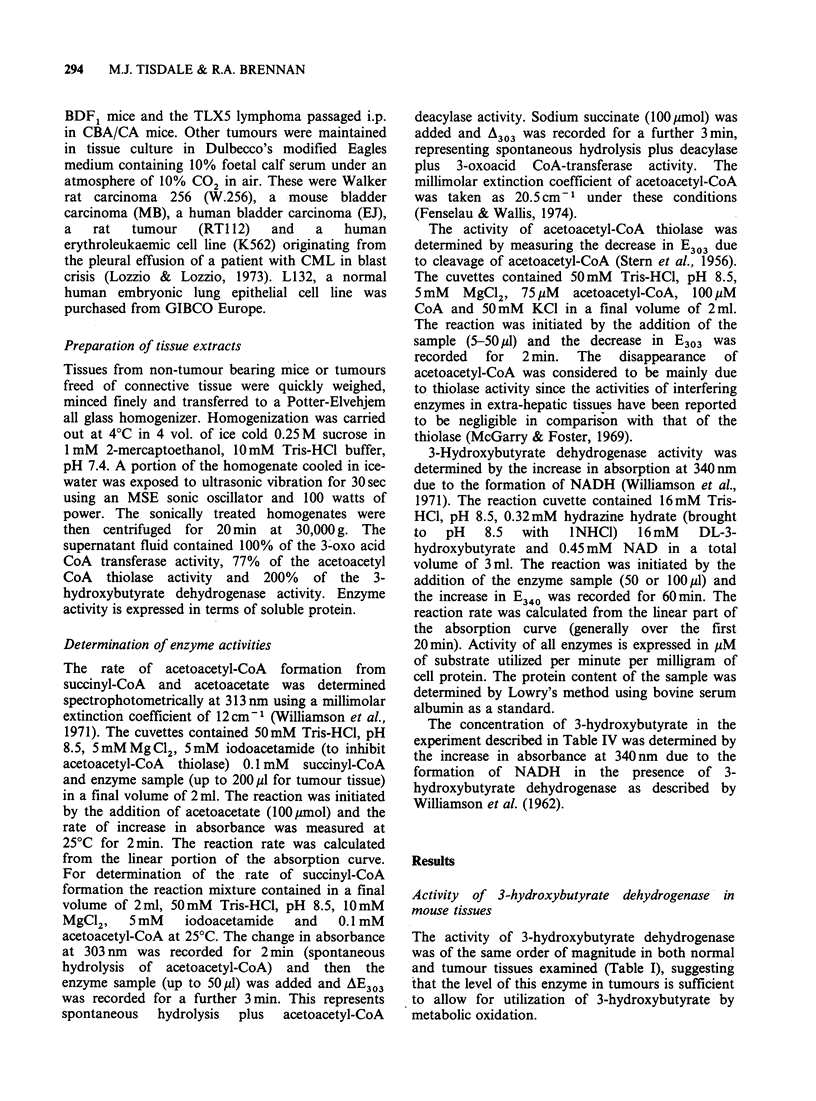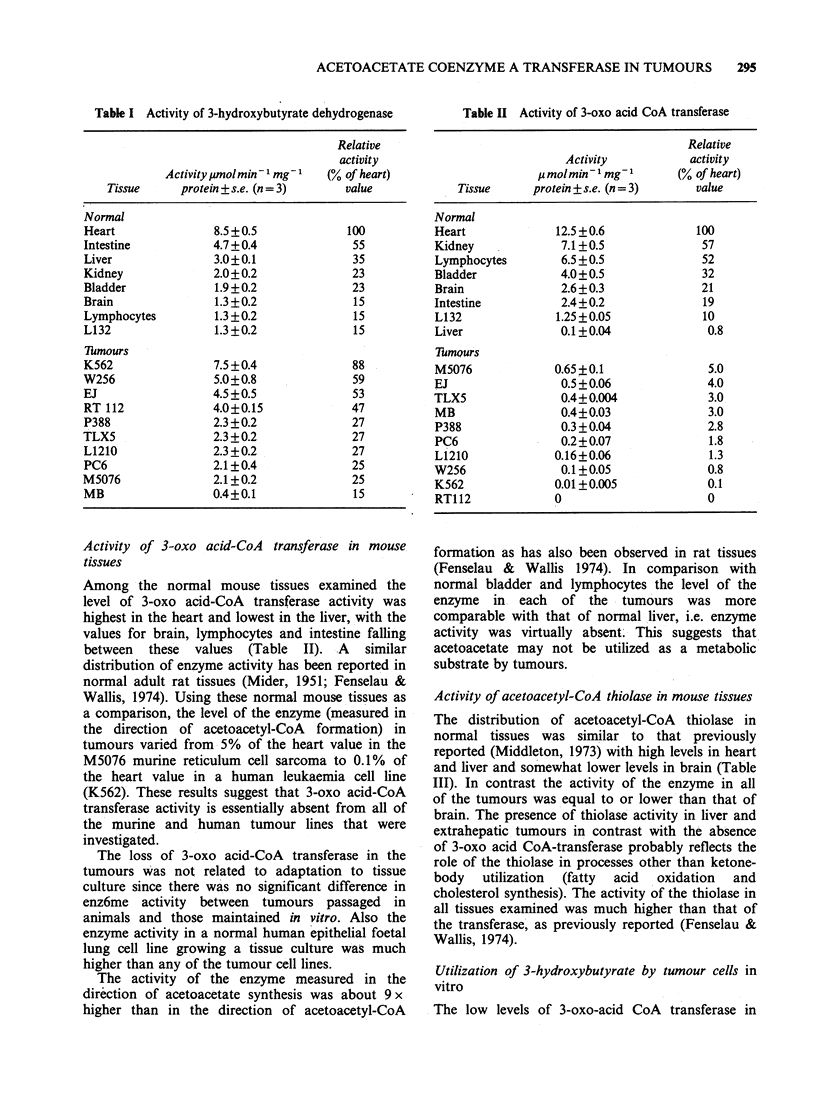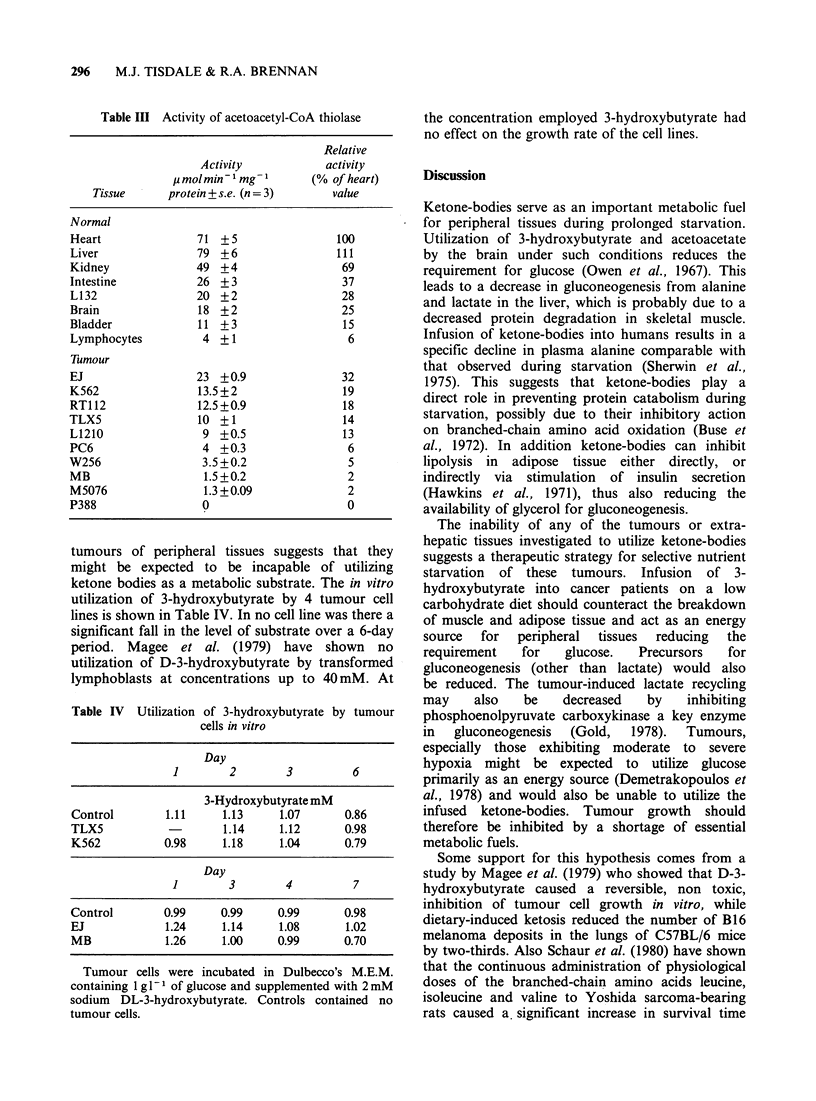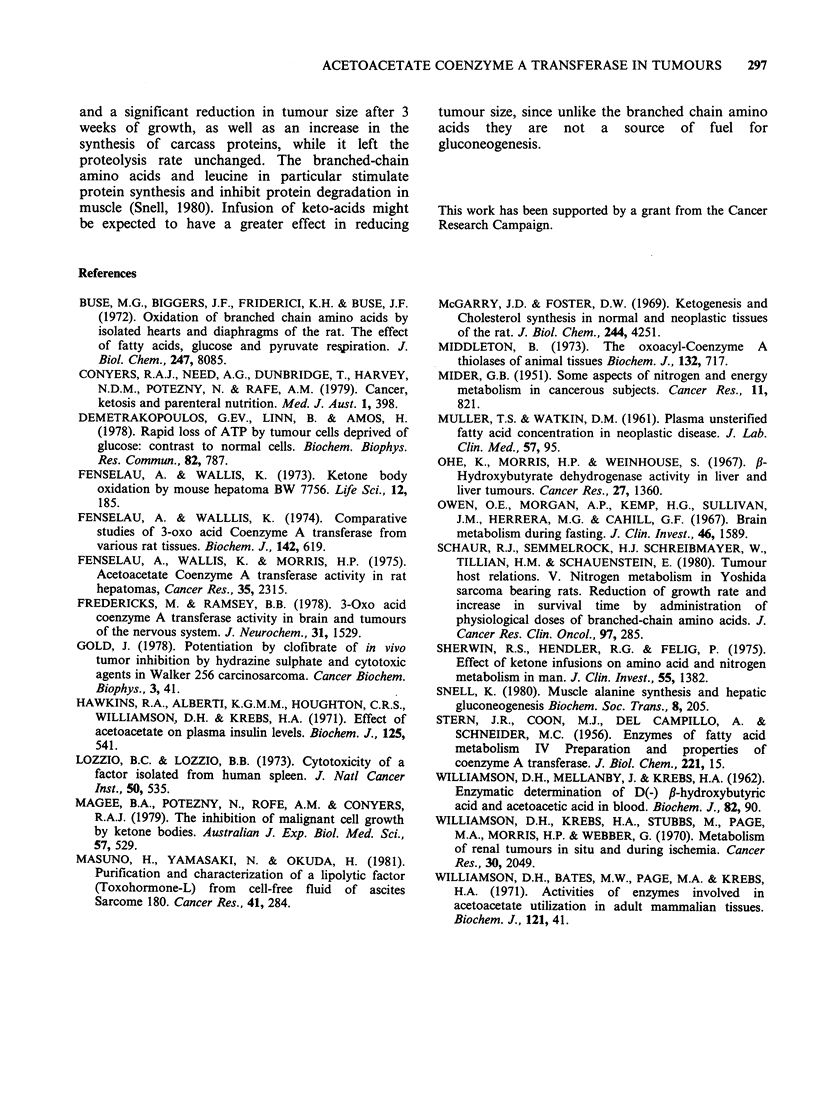# Loss of acetoacetate coenzyme A transferase activity in tumours of peripheral tissues.

**DOI:** 10.1038/bjc.1983.38

**Published:** 1983-02

**Authors:** M. J. Tisdale, R. A. Brennan

## Abstract

The presence of succinyl-coenzyme A: acetoacetate CoA--transferase (3-oxo acid-CoA transferase), an initiator of ketone body utilization in non-hepatic tissue was examined in a number of animal and human tumours of peripheral tissues. While enzyme levels in heart, kidney, lymphocytes and bladder were high, the tumours contained low or non-detectable levels of transferase activity, comparable with that of normal liver. The activities of acetoacetyl-CoA thiolase paralleled that of the transferase, except for the high activity in liver, and in all cases the tumour content of the enzyme was lower than that of the brain. The activity of 3-hydroxybutyrate dehydrogenase was similar in both normal and tumour tissue. The results indicate that tumours of non-hepatic tissues may be unable to metabolize ketone-bodies and suggest a therapeutic strategy for selective starvation of the tumour by dietary modification.


Br. J. Cancer (1983), 47, 293–297

### Loss of acetoacetate coenzyme A transferase activity in tumours of peripheral tissues

M.J. Tisdale & R.A. Brennan

*CRC Experimental Chemotherapy Group, Department of Pharmacy, University of Aston in Birmingham, Birmingham B4 7ET.*

**Summary** The presence of succinyl-coenzyme A: acetoacetate CoA—transferase (3-oxo acid-CoA transferase), an initiator of ketone body utilization in non-hepatic tissue was examined in a number of animal and human tumours of peripheral tissues. While enzyme levels in heart, kidney, lymphocytes and bladder were high, the tumours contained low or non-detectable levels of transferase activity, comparable with that of normal liver. The activities of acetoacetyl-CoA thiolase paralleled that of the transferase, except for the high activity in liver, and in all cases the tumour content of the enzyme was lower than that of the brain. The activity of 3-hydroxybutyrate dehydrogenase was similar in both normal and tumour tissue. The results indicate that tumours of non-hepatic tissues may be unable to metabolize ketone-bodies and suggest a therapeutic strategy for selective starvation of the tumour by dietary modification.

Hyperlipemia associated with mobilization of free fatty acids (FFA) from adipose tissue is a frequent companion of neoplastic diseases (Muller & Watkin, 1961). In certain cases the increased mobilization of FFA is probably due to a lipolytic factor (toxohormone L) produced by the tumour tissue (Masuno *et al.*, 1981). Despite the hyperlipemia ketonuria is an uncommon phenomenon in cancer cachexia (Mider, 1951; Conyers *et al.*, 1979). In starved tumour-bearing animals ketonemia is less than in controls and the utilization of ketone bodies by peripheral tissues of tumour-bearing rats tends to be higher than in tissues from controls (Mider, 1951).

Utilization of acetoacetate as an energy source requires the presence of two enzymes succinyl-coenzyme A: acetoacetate CoA-transferase (3 oxo acid-CoA transferase) (EC 2.8.3.5) and 3 oxoacyl-CoA thiolase (EC 2.3.1.9). The former enzyme is essentially absent from normal liver, but is present in peripheral tissues in varying amounts (Fenselau & Wallis, 1974; Williamson *et al.*, 1971). In contrast to normal liver considerable amounts of 3-oxo acid CoA-transferase are present in neonatal liver and in hepatomas, with the increased activity correlating with an increased growth rate (Fenselau & Wallis, 1973; Fenselau *et al.*, 1975). Such an enzyme shift could provide a metabolic advantage for the tumour. The tumour content of the enzyme required for the conversion of 3-hydroxybutyrate to acetoacetate, 3-hydroxybutyrate dehydrogenase (EC 1.1.1.30), however, has been found to decrease with increasing tumour growth rate, implying a possible limitation in tumour capacity for utilization of ketone bodies (Ohe *et al.*, 1967). The activity of 3-hydroxybutyrate dehydrogenase in renal tumours has been found to be considerably reduced in comparison with normal kidney whilst the levels of 3-oxo acid-CoA transferase and 3-oxoacyl-CoA thiolase in renal tumours were similar to that found in kidney (Williamson *et al.*, 1970). However, astrocytomas, glioblastoma multiforme, Schwannomas and craniopharyngiomas have been shown to contain considerably reduced 3 oxo acid-CoA transferase activity when compared with normal brain (Fredericks & Ramsey, 1978). The present study is designed to expand the knowledge on ketone body metabolism in tumour cells and reports the activities of 3 oxo acid-CoA transferase, acetoacetyl-CoA thiolase and 3-hydroxybutyrate dehydrogenase in a number of extra-hepatic animal and human tumours.

### Materials and methods

Acetoacetyl-CoA, succinyl-CoA, acetoacetate (lithium salt), sodium succinate, CoA and nicotinamide-adenine dinucleotides were purchased from Sigma Chemical Co. (Dorset) and were of the highest available purity. 3-Hydroxybutyrate dehydrogenase was obtained from Boehringer Corp. (London) Ltd.

*Tumours.*

The transplantable animal tumours used in this study were the PC6 plasmacytoma transplanted i.p. into Balb/c mice, P388 murine lymphocytic leukaemia and L1210 murine lymphocytic leukaemia transplanted i.p. into $BDF_1$ mice, M5076 murine reticulum cell sarcoma transplanted s.c. into

Received 6 August 1982; accepted 22 October 1982.

0007-0920/83/020293-05 $02.00

© The Macmillan Press Ltd., 1983

$BDF_1$ mice and the TLX5 lymphoma passaged i.p. in CBA/CA mice. Other tumours were maintained in tissue culture in Dulbecco's modified Eagles medium containing 10% foetal calf serum under an atmosphere of 10% $CO_2$ in air. These were Walker rat carcinoma 256 (W.256), a mouse bladder carcinoma (MB), a human bladder carcinoma (EJ), a rat tumour (RT112) and a human erythroleukaemic cell line (K562) originating from the pleural effusion of a patient with CML in blast crisis (Lozzio & Lozzio, 1973). L132, a normal human embryonic lung epithelial cell line was purchased from GIBCO Europe.

*Preparation of tissue extracts*

Tissues from non-tumour bearing mice or tumours freed of connective tissue were quickly weighed, minced finely and transferred to a Potter-Elvehjem all glass homogenizer. Homogenization was carried out at 4°C in 4 vol. of ice cold 0.25 M sucrose in 1 mM 2-mercaptoethanol, 10 mM Tris-HCl buffer, pH 7.4. A portion of the homogenate cooled in ice-water was exposed to ultrasonic vibration for 30 sec using an MSE sonic oscillator and 100 watts of power. The sonically treated homogenates were then centrifuged for 20 min at 30,000 g. The supernatant fluid contained 100% of the 3-oxo acid CoA transferase activity, 77% of the acetoacetyl CoA thiolase activity and 200% of the 3-hydroxybutyrate dehydrogenase activity. Enzyme activity is expressed in terms of soluble protein.

*Determination of enzyme activities*

The rate of acetoacetyl-CoA formation from succinyl-CoA and acetoacetate was determined spectrophotometrically at 313 nm using a millimolar extinction coefficient of 12 $cm^{-1}$ (Williamson *et al.*, 1971). The cuvettes contained 50 mM Tris-HCl, pH 8.5, 5 mM $MgCl_2$, 5 mM iodoacetamide (to inhibit acetoacetyl-CoA thiolase) 0.1 mM succinyl-CoA and enzyme sample (up to 200 $\mu$l for tumour tissue) in a final volume of 2 ml. The reaction was initiated by the addition of acetoacetate (100 $\mu$mol) and the rate of increase in absorbance was measured at 25°C for 2 min. The reaction rate was calculated from the linear portion of the absorption curve. For determination of the rate of succinyl-CoA formation the reaction mixture contained in a final volume of 2 ml, 50 mM Tris-HCl, pH 8.5, 10 mM $MgCl_2$, 5 mM iodoacetamide and 0.1 mM acetoacetyl-CoA at 25°C. The change in absorbance at 303 nm was recorded for 2 min (spontaneous hydrolysis of acetoacetyl-CoA) and then the enzyme sample (up to 50 $\mu$l) was added and $\Delta E_{303}$ was recorded for a further 3 min. This represents spontaneous hydrolysis plus acetoacetyl-CoA deacylase activity. Sodium succinate (100 $\mu$mol) was added and $\Delta_{303}$ was recorded for a further 3 min, representing spontaneous hydrolysis plus deacylase plus 3-oxoacid CoA-transferase activity. The millimolar extinction coefficient of acetoacetyl-CoA was taken as 20.5 $cm^{-1}$ under these conditions (Fenselau & Wallis, 1974).

The activity of acetoacetyl-CoA thiolase was determined by measuring the decrease in $E_{303}$ due to cleavage of acetoacetyl-CoA (Stern *et al.*, 1956). The cuvettes contained 50 mM Tris-HCl, pH 8.5, 5 mM $MgCl_2$, 75 $\mu$M acetoacetyl-CoA, 100 $\mu$M CoA and 50 mM KCl in a final volume of 2 ml. The reaction was initiated by the addition of the sample (5–50 $\mu$l) and the decrease in $E_{303}$ was recorded for 2 min. The disappearance of acetoacetyl-CoA was considered to be mainly due to thiolase activity since the activities of interfering enzymes in extra-hepatic tissues have been reported to be negligible in comparison with that of the thiolase (McGarry & Foster, 1969).

3-Hydroxybutyrate dehydrogenase activity was determined by the increase in absorption at 340 nm due to the formation of NADH (Williamson *et al.*, 1971). The reaction cuvette contained 16 mM Tris-HCl, pH 8.5, 0.32 mM hydrazine hydrate (brought to pH 8.5 with 1NHCl) 16 mM DL-3-hydroxybutyrate and 0.45 mM NAD in a total volume of 3 ml. The reaction was initiated by the addition of the enzyme sample (50 or 100 $\mu$l) and the increase in $E_{340}$ was recorded for 60 min. The reaction rate was calculated from the linear part of the absorption curve (generally over the first 20 min). Activity of all enzymes is expressed in $\mu$M of substrate utilized per minute per milligram of cell protein. The protein content of the sample was determined by Lowry's method using bovine serum albumin as a standard.

The concentration of 3-hydroxybutyrate in the experiment described in Table IV was determined by the increase in absorbance at 340 nm due to the formation of NADH in the presence of 3-hydroxybutyrate dehydrogenase as described by Williamson *et al.* (1962).

### Results

*Activity of 3-hydroxybutyrate dehydrogenase in mouse tissues*

The activity of 3-hydroxybutyrate dehydrogenase was of the same order of magnitude in both normal and tumour tissues examined (Table I), suggesting that the level of this enzyme in tumours is sufficient to allow for utilization of 3-hydroxybutyrate by metabolic oxidation.

**Table I** Activity of 3-hydroxybutyrate dehydrogenase

| *Tissue* | *Activity µmol min⁻¹ mg⁻¹ protein ± s.e. (n = 3)* | *Relative activity (% of heart) value* |
|---|---|---|
| *Normal* | | |
| Heart | 8.5 ± 0.5 | 100 |
| Intestine | 4.7 ± 0.4 | 55 |
| Liver | 3.0 ± 0.1 | 35 |
| Kidney | 2.0 ± 0.2 | 23 |
| Bladder | 1.9 ± 0.2 | 23 |
| Brain | 1.3 ± 0.2 | 15 |
| Lymphocytes | 1.3 ± 0.2 | 15 |
| L132 | 1.3 ± 0.2 | 15 |
| *Tumours* | | |
| K562 | 7.5 ± 0.4 | 88 |
| W256 | 5.0 ± 0.8 | 59 |
| EJ | 4.5 ± 0.5 | 53 |
| RT 112 | 4.0 ± 0.15 | 47 |
| P388 | 2.3 ± 0.2 | 27 |
| TLX5 | 2.3 ± 0.2 | 27 |
| L1210 | 2.3 ± 0.2 | 27 |
| PC6 | 2.1 ± 0.4 | 25 |
| M5076 | 2.1 ± 0.2 | 25 |
| MB | 0.4 ± 0.1 | 15 |

**Table II** Activity of 3-oxo acid CoA transferase

| *Tissue* | *Activity µmol min⁻¹ mg⁻¹ protein ± s.e. (n = 3)* | *Relative activity (% of heart) value* |
|---|---|---|
| *Normal* | | |
| Heart | 12.5 ± 0.6 | 100 |
| Kidney | 7.1 ± 0.5 | 57 |
| Lymphocytes | 6.5 ± 0.5 | 52 |
| Bladder | 4.0 ± 0.5 | 32 |
| Brain | 2.6 ± 0.3 | 21 |
| Intestine | 2.4 ± 0.2 | 19 |
| L132 | 1.25 ± 0.05 | 10 |
| Liver | 0.1 ± 0.04 | 0.8 |
| *Tumours* | | |
| M5076 | 0.65 ± 0.1 | 5.0 |
| EJ | 0.5 ± 0.06 | 4.0 |
| TLX5 | 0.4 ± 0.004 | 3.0 |
| MB | 0.4 ± 0.03 | 3.0 |
| P388 | 0.3 ± 0.04 | 2.8 |
| PC6 | 0.2 ± 0.07 | 1.8 |
| L1210 | 0.16 ± 0.06 | 1.3 |
| W256 | 0.1 ± 0.05 | 0.8 |
| K562 | 0.01 ± 0.005 | 0.1 |
| RT112 | 0 | 0 |

*Activity of 3-oxo acid-CoA transferase in mouse tissues*

Among the normal mouse tissues examined the level of 3-oxo acid-CoA transferase activity was highest in the heart and lowest in the liver, with the values for brain, lymphocytes and intestine falling between these values (Table II). A similar distribution of enzyme activity has been reported in normal adult rat tissues (Mider, 1951; Fenselau & Wallis, 1974). Using these normal mouse tissues as a comparison, the level of the enzyme (measured in the direction of acetoacetyl-CoA formation) in tumours varied from 5% of the heart value in the M5076 murine reticulum cell sarcoma to 0.1% of the heart value in a human leukaemia cell line (K562). These results suggest that 3-oxo acid-CoA transferase activity is essentially absent from all of the murine and human tumour lines that were investigated.

The loss of 3-oxo acid-CoA transferase in the tumours was not related to adaptation to tissue culture since there was no significant difference in enz6me activity between tumours passaged in animals and those maintained *in vitro*. Also the enzyme activity in a normal human epithelial foetal lung cell line growing a tissue culture was much higher than any of the tumour cell lines.

The activity of the enzyme measured in the direction of acetoacetate synthesis was about 9 × higher than in the direction of acetoacetyl-CoA formation as has also been observed in rat tissues (Fenselau & Wallis 1974). In comparison with normal bladder and lymphocytes the level of the enzyme in each of the tumours was more comparable with that of normal liver, i.e. enzyme activity was virtually absent. This suggests that acetoacetate may not be utilized as a metabolic substrate by tumours.

*Activity of acetoacetyl-CoA thiolase in mouse tissues*

The distribution of acetoacetyl-CoA thiolase in normal tissues was similar to that previously reported (Middleton, 1973) with high levels in heart and liver and somewhat lower levels in brain (Table III). In contrast the activity of the enzyme in all of the tumours was equal to or lower than that of brain. The presence of thiolase activity in liver and extrahepatic tumours in contrast with the absence of 3-oxo acid CoA-transferase probably reflects the role of the thiolase in processes other than ketone-body utilization (fatty acid oxidation and cholesterol synthesis). The activity of the thiolase in all tissues examined was much higher than that of the transferase, as previously reported (Fenselau & Wallis, 1974).

*Utilization of 3-hydroxybutyrate by tumour cells in* vitro

The low levels of 3-oxo-acid CoA transferase in

**Table III** Activity of acetoacetyl-CoA thiolase

| *Tissue* | *Activity* $\mu mol\,min^{-1}\,mg^{-1}$ *protein* $\pm$ *s.e.* ($n=3$) | *Relative activity (% of heart) value* |
|---|---|---|
| *Normal* | | |
| Heart | 71 ±5 | 100 |
| Liver | 79 ±6 | 111 |
| Kidney | 49 ±4 | 69 |
| Intestine | 26 ±3 | 37 |
| L132 | 20 ±2 | 28 |
| Brain | 18 ±2 | 25 |
| Bladder | 11 ±3 | 15 |
| Lymphocytes | 4 ±1 | 6 |
| *Tumour* | | |
| EJ | 23 ±0.9 | 32 |
| K562 | 13.5±2 | 19 |
| RT112 | 12.5±0.9 | 18 |
| TLX5 | 10 ±1 | 14 |
| L1210 | 9 ±0.5 | 13 |
| PC6 | 4 ±0.3 | 6 |
| W256 | 3.5±0.2 | 5 |
| MB | 1.5±0.2 | 2 |
| M5076 | 1.3±0.09 | 2 |
| P388 | 0 | 0 |

tumours of peripheral tissues suggests that they might be expected to be incapable of utilizing ketone bodies as a metabolic substrate. The *in vitro* utilization of 3-hydroxybutyrate by 4 tumour cell lines is shown in Table IV. In no cell line was there a significant fall in the level of substrate over a 6-day period. Magee *et al.* (1979) have shown no utilization of D-3-hydroxybutyrate by transformed lymphoblasts at concentrations up to 40 mM. At the concentration employed 3-hydroxybutyrate had no effect on the growth rate of the cell lines.

**Table IV** Utilization of 3-hydroxybutyrate by tumour cells *in vitro*

| | *Day* 1 | 2 | 3 | 6 |
|---|---|---|---|---|
| | 3-Hydroxybutyrate mM | | | |
| Control | 1.11 | 1.13 | 1.07 | 0.86 |
| TLX5 | — | 1.14 | 1.12 | 0.98 |
| K562 | 0.98 | 1.18 | 1.04 | 0.79 |
| | *Day* 1 | 3 | 4 | 7 |
| Control | 0.99 | 0.99 | 0.99 | 0.98 |
| EJ | 1.24 | 1.14 | 1.08 | 1.02 |
| MB | 1.26 | 1.00 | 0.99 | 0.70 |

Tumour cells were incubated in Dulbecco's M.E.M. containing $1\,g\,l^{-1}$ of glucose and supplemented with 2 mM sodium DL-3-hydroxybutyrate. Controls contained no tumour cells.

### Discussion

Ketone-bodies serve as an important metabolic fuel for peripheral tissues during prolonged starvation. Utilization of 3-hydroxybutyrate and acetoacetate by the brain under such conditions reduces the requirement for glucose (Owen *et al.*, 1967). This leads to a decrease in gluconeogenesis from alanine and lactate in the liver, which is probably due to a decreased protein degradation in skeletal muscle. Infusion of ketone-bodies into humans results in a specific decline in plasma alanine comparable with that observed during starvation (Sherwin *et al.*, 1975). This suggests that ketone-bodies play a direct role in preventing protein catabolism during starvation, possibly due to their inhibitory action on branched-chain amino acid oxidation (Buse *et al.*, 1972). In addition ketone-bodies can inhibit lipolysis in adipose tissue either directly, or indirectly via stimulation of insulin secretion (Hawkins *et al.*, 1971), thus also reducing the availability of glycerol for gluconeogenesis.

The inability of any of the tumours or extra-hepatic tissues investigated to utilize ketone-bodies suggests a therapeutic strategy for selective nutrient starvation of these tumours. Infusion of 3-hydroxybutyrate into cancer patients on a low carbohydrate diet should counteract the breakdown of muscle and adipose tissue and act as an energy source for peripheral tissues reducing the requirement for glucose. Precursors for gluconeogenesis (other than lactate) would also be reduced. The tumour-induced lactate recycling may also be decreased by inhibiting phosphoenolpyruvate carboxykinase a key enzyme in gluconeogenesis (Gold, 1978). Tumours, especially those exhibiting moderate to severe hypoxia might be expected to utilize glucose primarily as an energy source (Demetrakopoulos *et al.*, 1978) and would also be unable to utilize the infused ketone-bodies. Tumour growth should therefore be inhibited by a shortage of essential metabolic fuels.

Some support for this hypothesis comes from a study by Magee *et al.* (1979) who showed that D-3-hydroxybutyrate caused a reversible, non toxic, inhibition of tumour cell growth *in vitro*, while dietary-induced ketosis reduced the number of B16 melanoma deposits in the lungs of C57BL/6 mice by two-thirds. Also Schaur *et al.* (1980) have shown that the continuous administration of physiological doses of the branched-chain amino acids leucine, isoleucine and valine to Yoshida sarcoma-bearing rats caused a significant increase in survival time and a significant reduction in tumour size after 3 weeks of growth, as well as an increase in the synthesis of carcass proteins, while it left the proteolysis rate unchanged. The branched-chain amino acids and leucine in particular stimulate protein synthesis and inhibit protein degradation in muscle (Snell, 1980). Infusion of keto-acids might be expected to have a greater effect in reducing tumour size, since unlike the branched chain amino acids they are not a source of fuel for gluconeogenesis.

This work has been supported by a grant from the Cancer Research Campaign.

### References

BUSE, M.G., BIGGERS, J.F., FRIDERICI, K.H. & BUSE, J.F. (1972). Oxidation of branched chain amino acids by isolated hearts and diaphragms of the rat. The effect of fatty acids, glucose and pyruvate respiration. *J. Biol. Chem.*, **247,** 8085.

CONYERS, R.A.J., NEED, A.G., DUNBRIDGE, T., HARVEY, N.D.M., POTEZNY, N. & RAFE, A.M. (1979). Cancer, ketosis and parenteral nutrition. *Med. J. Aust.* **1,** 398.

DEMETRAKOPOULOS, G.EV., LINN, B. & AMOS, H. (1978). Rapid loss of ATP by tumour cells deprived of glucose: contrast to normal cells. *Biochem. Biophys. Res. Commun.*, **82,** 787.

FENSELAU, A. & WALLIS, K. (1973). Ketone body oxidation by mouse hepatoma BW 7756. *Life Sci.*, **12,** 185.

FENSELAU, A. & WALLLIS, K. (1974). Comparative studies of 3-oxo acid Coenzyme A transferase from various rat tissues. *Biochem. J.*, **142,** 619.

FENSELAU, A., WALLIS, K. & MORRIS, H.P. (1975). Acetoacetate Coenzyme A transferase activity in rat hepatomas, *Cancer Res.*, **35,** 2315.

FREDERICKS, M. & RAMSEY, B.B. (1978). 3-Oxo acid coenzyme A transferase activity in brain and tumours of the nervous system. *J. Neurochem.*, **31,** 1529.

GOLD, J. (1978). Potentiation by clofibrate of *in vivo* tumor inhibition by hydrazine sulphate and cytotoxic agents in Walker 256 carcinosarcoma. *Cancer Biochem. Biophys.*, **3,** 41.

HAWKINS, R.A., ALBERTI, K.G.M.M., HOUGHTON, C.R.S., WILLIAMSON, D.H. & KREBS, H.A. (1971). Effect of acetoacetate on plasma insulin levels. *Biochem. J.*, **125,** 541.

LOZZIO, B.C. & LOZZIO, B.B. (1973). Cytotoxicity of a factor isolated from human spleen. *J. Natl Cancer Inst.*, **50,** 535.

MAGEE, B.A., POTEZNY, N., ROFE, A.M. & CONYERS, R.A.J. (1979). The inhibition of malignant cell growth by ketone bodies. *Australian J. Exp. Biol. Med. Sci.*, **57,** 529.

MASUNO, H., YAMASAKI, N. & OKUDA, H. (1981). Purification and characterization of a lipolytic factor (Toxohormone-L) from cell-free fluid of ascites Sarcome 180. *Cancer Res.*, **41,** 284.

McGARRY, J.D. & FOSTER, D.W. (1969). Ketogenesis and Cholesterol synthesis in normal and neoplastic tissues of the rat. *J. Biol. Chem.*, **244,** 4251.

MIDDLETON, B. (1973). The oxoacyl-Coenzyme A thiolases of animal tissues *Biochem. J.*, **132,** 717.

MIDER, G.B. (1951). Some aspects of nitrogen and energy metabolism in cancerous subjects. *Cancer Res.*, **11,** 821.

MULLER, T.S. & WATKIN, D.M. (1961). Plasma unsterified fatty acid concentration in neoplastic disease. *J. Lab. Clin. Med.*, **57,** 95.

OHE, K., MORRIS, H.P. & WEINHOUSE, S. (1967). β-Hydroxybutyrate dehydrogenase activity in liver and liver tumours. *Cancer Res.*, **27,** 1360.

OWEN, O.E., MORGAN, A.P., KEMP, H.G., SULLIVAN, J.M., HERRERA, M.G. & CAHILL, G.F. (1967). Brain metabolism during fasting. *J. Clin. Invest.*, **46,** 1589.

SCHAUR, R.J., SEMMELROCK, H.J. SCHREIBMAYER, W., TILLIAN, H.M. & SCHAUENSTEIN, E. (1980). Tumour host relations. V. Nitrogen metabolism in Yoshida sarcoma bearing rats. Reduction of growth rate and increase in survival time by administration of physiological doses of branched-chain amino acids. *J. Cancer Res. Clin. Oncol.*, **97,** 285.

SHERWIN, R.S., HENDLER, R.G. & FELIG, P. (1975). Effect of ketone infusions on amino acid and nitrogen metabolism in man. *J. Clin. Invest.*, **55,** 1382.

SNELL, K. (1980). Muscle alanine synthesis and hepatic gluconeogenesis *Biochem. Soc. Trans.*, **8,** 205.

STERN, J.R., COON, M.J., DEL CAMPILLO, A. & SCHNEIDER, M.C. (1956). Enzymes of fatty acid metabolism IV Preparation and properties of coenzyme A transferase. *J. Biol. Chem.*, **221,** 15.

WILLIAMSON, D.H., MELLANBY, J. & KREBS, H.A. (1962). Enzymatic determination of D(-) β-hydroxybutyric acid and acetoacetic acid in blood. *Biochem. J.*, **82,** 90.

WILLIAMSON, D.H., KREBS, H.A., STUBBS, M., PAGE, M.A., MORRIS, H.P. & WEBBER, G. (1970). Metabolism of renal tumours in situ and during ischemia. *Cancer Res.*, **30,** 2049.

WILLIAMSON, D.H., BATES, M.W., PAGE, M.A. & KREBS, H.A. (1971). Activities of enzymes involved in acetoacetate utilization in adult mammalian tissues. *Biochem. J.*, **121,** 41.